# Genetic Modification of Limbal Stem Cells to Decrease Allogeneic Immune Responses

**DOI:** 10.3389/fimmu.2021.747357

**Published:** 2021-12-09

**Authors:** Emilio Valdivia, Marina Bertolin, Claudia Breda, Marco Carvalho Oliveira, Anna Katharina Salz, Nicola Hofmann, Martin Börgel, Rainer Blasczyk, Stefano Ferrari, Constanca Figueiredo

**Affiliations:** ^1^ Institute of Transfusion Medicine and Transplant Engineering, Hannover, Germany; ^2^ Fondazione Banca degli Occhi del Veneto, Venice, Italy; ^3^ German Society for Tissue Transplantation (DGFG), Hannover, Germany

**Keywords:** limbal stem cell, RNA interference, HLA, limbal stem cell deficiency, allotransplantation, lentiviral vector, gene therapy

## Abstract

Limbal stem cell (LSC) transplantation is the only efficient treatment for patients affected by LSC deficiency (LSCD). Allogeneic LSC transplantation is one of the most successful alternative for patients with bilateral LSCD. Nevertheless, the high variability of the human leukocyte antigens (HLA) remains a relevant obstacle to long-term allogeneic graft survival. This study characterized the immunologic properties of LSCs and proposed a genetic engineering strategy to reduce the immunogenicity of LSCs and of their derivatives. Hence, LSC HLA expression was silenced using lentiviral vectors encoding for short hairpin (sh) RNAs targeting β2-microglobulin (β2M) or class II major histocompatibility complex transactivator (CIITA) to silence HLA class I and II respectively. Beside the constitutive expression of HLA class I, LSCs showed the capability to upregulate HLA class II expression under inflammatory conditions. Furthermore, LSCs demonstrated the capability to induce T-cell mediated immune responses. LSCs phenotypical and functional characteristics are not disturbed after genetic modification. However, HLA silenced LSC showed to prevent T cell activation, proliferation and cytotoxicity in comparison to fully HLA-expressing LSCs. Additionally; HLA-silenced LSCs were protected against antibody-mediated cellular-dependent cytotoxicity. Our data is a proof-of-concept of the feasibility to generate low immunogenic human LSCs without affecting their typical features. The use of low immunogenic LSCs may support for long-term survival of LSCs and their derivatives after allogeneic transplantation.

## Introduction

Limbal stem cells (LSCs) constantly maintain the homeostasis of corneal epithelium. LSCs are located in the palisades of Vogt and give origin to transient amplifying cells (TACs), which migrate to the central part of the cornea to differentiate into epithelial cells (1). LSC deficiency (LSCD) is a pathological condition characterized by the loss of LSCs (2). The main causes leading to LSCD are chemical and thermal burns, ultraviolet exposure, ionizing radiation, chemotherapeutic agents, viral, bacterial or fungal infections or genetic disorders (3). LSCD treatment depends on the cause and severity of the injury (2). Therapeutic options range from non-surgical such as autologous serum eye drops or therapeutic lens to surgical interventions with the application of amniotic membrane, keratolimbal allograft (KLAL), simple limbal epithelial transplantation (SLET), conjunctival limbal allograft (CLAL) or cultivated LSCs transplantation (CLET) (4–8). CLET is one of the most effective showing 70 to 80% success rate in the regeneration of the corneal epithelium (9). Pellegrini et al. first described autologous CLET in which limbal epithelial cells from the patient´s healthy eye were collected, cultivated and expanded *in vitro* prior transplantation into the diseased eye (10). Allogeneic CLET (allo-CLET) relies on cells from living donors or cadaveric limbal tissues. However, allo-CLET is associated with continuous systemic immunosuppression to prevent allograft loss (11). Unfortunately, adverse effects related to immunosuppression regimes are always present and demand closed monitoring (12).

Even though the ocular microenvironment is known to be immune privileged (13), neovascularization associated with LSCD, previous treatments and surgery itself often destroy the immune privilege increasing the susceptibility of the graft to strong alloimmune response and rejection (14, 15). Despite some concerns of allo-CLET regarding immunosuppression, rejection, culture techniques and others; one of the clear advantages of allo-CLET is the amount of initial tissue for cultivation that can be used and its quality, aspects that has been described to influence transplantation success (9).

CLET technique gives the opportunity to genetically engineer LSCs either to correct autologous malignancies or to improve graft survival by minimizing graft’s immunogenicity. Expression of HLA class I and II on LSCs directly or on the derived epithelial cells can trigger allogeneic T-cell mediated immune response leading to the rejection of the graft (16). Indeed, the high variability of HLA is one of the greatest obstacles for long term allograft survival (17).

LSCs are known to express constitutively HLA class I, but not HLA class II molecules. However, their immunological properties and immunogenic potential remain unclear. Furthermore, after transplantation, LSCs are expected to restore the corneal dynamic equilibrium by producing TACs and terminal differentiated cells (TDCs). These last cells have been described to be able to upregulate HLA class I and II molecules disrupting immune privilege and contributing to immune rejection (18–20).

Downregulation of HLA class I and class II molecules has been observed to prevent *de novo* and pre-formed alloimmune response (21, 22). Previously, we have shown that the downregulation of HLA class I and II expression in different cell types generates an immune invisible state in which cells are protected from rejection after allotransplantation.

In this study, we characterized the immunological properties of LSCs and investigated the possibility to genetically engineer them towards reduction of their immunogenicity without affecting their phenotypic and functional properties.

## Materials and Methods

### Limbal Stem Cell Isolation and Culture

Human limbal tissues were harvested from post-mortem donation corneas not suitable for transplantation. Donor corneas were used for research purposes after a written consent was obtained by the donor’s next of kin following the guidelines of the Italian Transplant Centre (CNT, Rome, Italy), the guidelines of the German Medical Association for the collection of donor corneas and managing an eye bank, as well as the Tenets of the Declaration of Helsinki. LSCs were cultured as previously described (23). Briefly, limbal rims were isolated and treated with 3-4 cycles of trypsin digestion at 37°C for 30 min. Isolated LSCs were plated at a seeding concentration of 20 000/cm2 onto lethally irradiated 3T3 fibroblasts using DMEM (Gibco, Massachusetts, USA) supplemented with F-12 Nutrient Mixture (Gibco), 10% fetal bovine serum (Gibco), and supplemented with 4 mM L-glutamine (C.C pro, Oberdorla, Germany), 0.18 mM adenine (Sigma‐aldrich, Missouri, USA), 0.4 μg/mL hydrocortisone (MERCK, Darmstadt, Germany), 5 μg/mL insulin (Sigma‐aldrich), 2 nM triiodothyronine (Sigma‐aldrich), 8.1 μg/mL cholera toxin (Sigma‐aldrich), 10 ng/mL recombinant human epidermal growth factor (EGF) (Peprotech, Hamburg, Germany), and 2% penicillin/streptomycin (Gibco). The medium was changed every other day and LSCs maintained at 37°C in 5% CO_2_ humidified atmosphere.

### Vector Production and LSCs Transduction

Lentiviral vectors encoding for the GFP gene sequence as reporter and encoding for β2M- or CIITA-specific shRNAs, or a control non-sense shRNA encoding vector were produced by transfection of HEK-293T cells in HYPERFlask^®^ vessels (Corning, Darmstadt, Germany). For lentiviral vector particle production, HEK-293T cells were cotransfected with the shRNA-sequence encoding plasmid, lentiviral packaging plasmid (psPAX2) and VSV-G envelope expressing plasmid (pMD2.G) using polyethylenimine. After 48 hours, vector-containing culture supernatant was collected, filtered and centrifuged for 3 hours at 20000 rpm at 16°C. Vector pellets were resuspended in LSCs medium and stored at -80°C.

Approximately 2x10^6^ LSCs were transduced in presence of protamine sulphate (Sigma‐Aldrich, Missouri, USA), as previously described (24, 25). Next day, LSCs were enzymatically detached and seeded onto feeder cells with fresh supplemented LSC medium.

### Transcripts Levels Analysis

Total RNA was isolated from LSCs (RNeasy Mini Kit, Qiagen, Hilden, Germany) and reverse transcribed to cDNA using the high‐capacity cDNA reverse transcription kit (Applied Biosystems, Darmstadt, Germany). Transcripts levels of β2M, CIITA, HLA-DR, ABCB5, p63α and CK12 were analyzed by qPCR using specific predesigned TaqMan Gene Expression Assays (HS00984230_m1, Hs00932860_m1, Hs00219575_m1, Hs02889060_m1, Hs00978344_m1, Hs00165015_m1, respectively; Thermo Fisher, Massachusetts, USA). Samples were analyzed in triplicates and target gene levels were normalized to glyceraldehyde‐3‐phosphate dehydrogenase (GAPDH) (Hs02758991_g1) (Thermo Fisher).

### Flow Cytometry

LSC were carefully detached from culture plates using TrypLE (Thermo Fisher, Massachusetts, USA) and incubated for surface marker detection with anti-HLA-ABC PE-conjugated antibodies (clone W6/32; Biorad, California, USA) and anti-HLA-DR APC/Cy7-conjugated antibodies (clone L243; Biolegend, California, USA). ABCB5 protein detection was performed with an anti-ABCB5 unconjugated antibody (polyclonal; Thermo Fisher Scientific) and a PE-conjugated secondary antibody. For intracellular markers, LSCs were permeabilized (IntraPrep Permabilization Kit, Beckman Coulter, Krefeld, Germany) and stained with primary antibodies specific for p63α (clone I504; Abbexa, Cambridge, UK) and CK12 (clone EPR17882; Abcam, Cambridge, UK) followed by PE- and APC/Cy7- conjugated secondary antibodies, respectively. Transduction efficiency was evaluated by detecting GFP expression. Data acquisition was performed using a FACSCanto II Flow Cytometer (Becton, Dickinson & Company, New Jersy, USA) and the results were analyzed using FlowJo software (Becton, Dickinson & Company). Evaluation of anti-human antibodies cross-reactivity was performed after staining 3T3 feeders cells (**Supplementary Figure 1**).

### Antibody-Dependent Cell-Mediated Cytotoxicity Assay

ADCC Reporter Bioassay core Kit (Promega, Wisconsin, USA) was used following manufacturer’s instructions. Briefly, silenced and non- silenced LSCs were stimulated with interferon (IFN)ɣ (100ng/mL) for 48 hours. Twenty four hours before the assay, 1.25x10^4^ LSCs were seeded in each well of a 96-well plate. At the day of the assay, antibodies specific for human HLA-ABC and HLA-DR were added. Afterwards, LSCs were incubated in the presence of effector cells (Ratio 1:6, T: E) provided in the kit for 6 hours at 37°C. Cell activation rates were evaluated by adding Bio-Glo™ Luciferase assay substrate reagent to the cultures and luminescence was measured using a Synergy 2 Multi-Detection Microplate Reader (Biotek, Winooski, USA).

### T Lymphocyte Proliferation Assay

Human T cells were isolated from healthy donors. First, peripheral blood mononuclear cells were isolated from whole blood by density gradient centrifugation using Lymphosep (C.C pro, Oberdorla, Germany). Negative T cell isolation was performed with the Pan T cell kit (Miltenyi Biotech, Bergisch Gladbach, Germany) following the manufacturer’s instructions. T cells were stained with the cell proliferation dye efluor 670 (Thermo Fischer Scientific, Massachusetts, USA) and exposed to HLA-silenced and non-silenced LSCs which had been previously stimulated for 48 hours with IFNɣ (100ng/mL). Proliferation assay was performed using a target: effector ratio of 1:3 in RPMI 1640 supplemented with 5% AB serum and interleukin (IL)-2 (100U/ml) (Prepotech, New Jersey, USA) for 7 days. At day 5 of the proliferation assay, T cells were re-stimulated with the same native or genetically engineered LSC targets also previously treated with IFNɣ (100ng/mL). After 7 days of experiment, T cell proliferation rates were analyzed by flow cytometry.

### Real Time Cytotoxicity Assay (Cell Index)

All experiments were performed with T cells isolated from healthy donors as described above. Isolated T cells were primed with fully HLA-expressing LSCs which were previously stimulated with IFNɣ (100ng/mL) for 48 hours. T cell priming was performed for 7 days in RPMI 1640 Medium (Lonza, Basel, Switzerland) supplemented with 5% AB serum and IL-2 (100U/ml) (Prepotech, New Jersey, USA), IL-7 (100ng/mL) (Prepotech, New Jersey, USA) and IL-12 (50ng/mL) (Prepotech, New Jersey, USA). Real time cytotoxicity was measured using XCelligence RTCA DP analyzer (Agilent Technologies, California, USA). First, background signal was measured using E-plate 16 (Agilent Technologies, California, USA) with 50µl medium. Silenced and non-silenced LSCs (target cells) were seeded and let adhere for 24 hours. Subsequently, pre-stimulated allogeneic T cells (effector cells) were added in a 1:2 ratio (target: effector). Changes in electrical impedance were expressed as cell index values, which correlates with cellular coverage of electrode sensors at the bottom of each well, and normalized to baseline impedance values before adding effector cells.

### NK Cell Co-Culture With LSCs

NK cells were isolated from healthy donors by negative selection using magnetic‐activated cell sorting (Miltenyi Biotec Bergisch). Isolated NK cells were maintained overnight in RPMI-1640 (Lonza, Basel, Switzerland) supplemented with 5% human serum (C.C pro, Oberdorla, Germany) and IL‐2 (100U/mL) (Prepotech, New Jersey, USA). HLA-silenced and non-silenced LSCs unstimulated or IFNɣ-stimulated (100ng/mL) for 48 hours were used to evaluate NK cell degranulation. At the day of the experiment, LSCs were cultured with NK cells in a target: effector ratio of 1:2 in 96-well plates (Falcon, Corning Brand) for 5 hours at 37°C. After incubation time, NK cells were collected, stained with anti- CD3-APC/Cy7 (clone: HIT3a; Biolegend, California, USA), anti-CD56-AF700 (clone: B159; BD Biosciences, New Jersey, USA) and CD107a-PE (clone: H4A3; Biolegend, California, USA) and analyzed by flow cytometry. NK cell activation was evaluated by measuring CD107a expression as a marker of degranulation.

### Cytokine Multiplex Analyses

T cells isolated from peripheral blood of healthy donors were pre-stimulated (primed) by incubating them with fully HLA-expressing LSCs which were previously treated with IFNɣ (100ng/mL) for 48 hours. Priming was performed in RPMI 1640 Medium (Lonza, Basel, Switzerland) supplemented with 5% AB serum and 100U/mL of IL-2 (Prepotech, New Jersey, USA), 100ng/mL of IL-7 (Prepotech) and 50ng/mL of IL-12 (Prepotech) for 7 days. To perform the assay, β2M-, CIITA-silenced LSCs or non-silenced LSCs previously treated with IFNɣ (100ng/mL) for 48 hours were incubated with primed T-cells in a ratio target: effector 1:5 for 6 hours in RPMI 1640 Medium (Lonza, Basel, Switzerland) supplemented with 5% AB serum and IL-2 (100U/mL). Cell culture supernatants were collected and analyzed for cytokines secretion of IL-5, IL-6, IL-8, IL-10, IL-17A, granulocytes-macrophage colony-stimulating factor (GM-CSF), IFNγ, IFNɣ-induced protein 10 (IP-10), and tumor necrosis factor (TNF)α using MILLIPLEX^®^ Map Human Cytokine/Chemokine Magnetic Bead Panel (Merck Millipore, Darmstadt, Germany) and a Luminex^®^ 100/200™ analyzer (Luminex Corp., Texas, USA) according to manufacturer’s instructions. Cytokine concentrations were calculated using the Xponent software version 3.1 (Thermo Fischer, Massachusetts, USA).

### Statistical Analysis

Data were analyzed using GraphPad Prim 8 software (GraphPad Software, San Diego, USA). Results are shown as mean ± standard deviation (SD). One-way ANOVA or student t test were used to compare differences between groups. Level of significance was set at p<0.05 (*p<0.05, **p<0.01, ***p<0.001, ****p<0.0001).

## Results

### Pro-Inflammatory Conditions Induce Upregulation of HLA Expression on Limbal Stem Cells

Immediately after transplantation, LSCs are subjected to an inflammatory microenvironment due to transplantation surgery itself. Under such circumstances, allogeneic LSCs and their mature cell progeny are expected to respond to this inflammatory microenvironment. One of the cytokines playing a relevant role in inflammation is IFNɣ, which is known to induce upregulation of MHC expression (26). Here, we used 48 hours stimulation with IFNɣ (100ng/mL) to mimic an inflammatory microenvironment.

**Figure 1:**
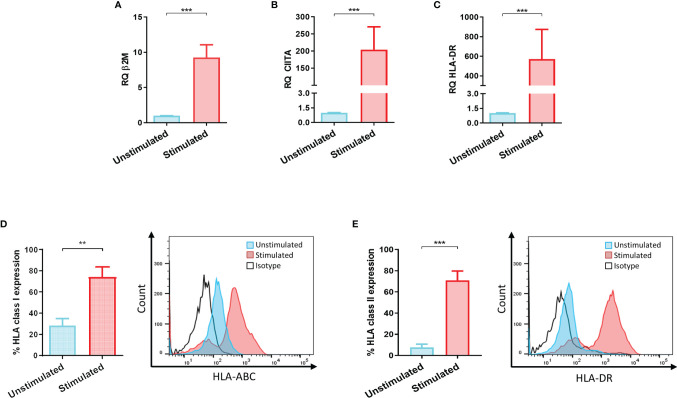
Inflammatory microenvironment promotes upregulation of HLA antigens on limbal stem cells. **(A)** HLA class I codominant domain β2-microglobulin (β2M), **(B)** HLA class II transactivator (CIITA) and **(C)** HLA-DR transcripts upregulated on limbal stem cells (LSCs) after 48 hours stimulation with IFNγ (100ng/mL). **(D, E)** Surface mean expression of HLA class I and class II molecules on LSCs in absence and presence of IFNγ stimulation. Student’s t-test was used to compared differences between groups and data are presented as mean ± SD, n = 5, **p < 0.01, ***p < 0.001.

LSCs were stimulated with IFNγ to evaluate their capacity to upregulate HLA class I and class II levels. β2M, CIITA and HLA-DR transcripts as well as surface expression of HLA class I and II proteins were analyzed on stimulated and non-stimulated LSCs (**Figures 1A**–**C**). After IFNγ stimulation, transcript level analyses showed an upregulation of 9-, 200-, 500 folds of β2m, CIITA and HLA-DR, respectively. These data correlated with an increase of HLA class I and II surface expression compared to unstimulated LSCs. Expression of cell surface HLA class I molecules on LSCs was 2-fold higher (unstimulated: 28.4% ± 14.3%, stimulated: 74.1% ± 21.5%, p<0.01) after exposure to IFNγ. In the case of HLA class II molecules, their surface expression was 10-fold higher than unstimulated LSCs (unstimulated: 7.6% ± 6.3%; stimulated: 71.0% ± 17.6%, p<0.001) (**Figures 1D, E**).

### Limbal Stem Cell Morphology and Growth Dynamics Are Not Compromised After Viral Transduction and HLA Silencing

**Figure 2:**
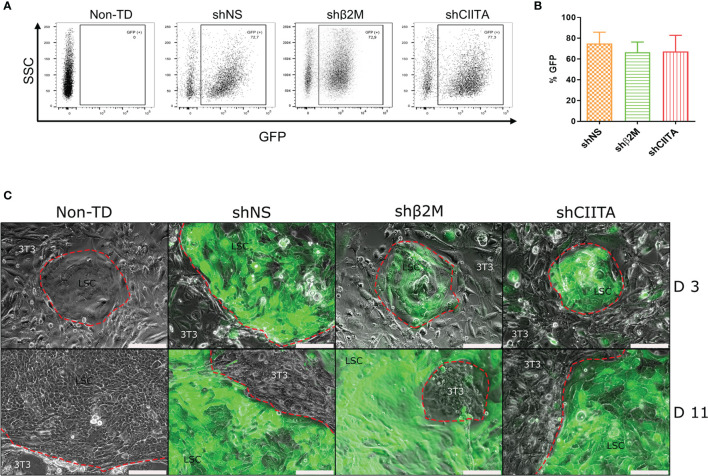
Limbal stem cell morphology and growth dynamics are not compromised after lentviral transduction and silencing HLA expression. **(A)** GFP expression of transduced cells with shNS-, shβ2M- or shCIITA-encoding vectors. **(B)** Mean GFP expression of transduced LSCs (n=6). Data are presented as mean ± SD.**(C)** Limbal stem cells (LSCs) cultivated onto lethally irradiated 3T3 feeder cells at day 3 (d3) and day 11 (d11) after transduction with shNS (vector control), shβ2M (shRNA targeting β2-microglobulin) and shCIITA (shRNA targeting class II major histocompatibility complex transactivator). LSCs form typical colonies with preserved small stemness-like-morphology. Scale bar: 200µm.

An efficient transduction is the key to ensure LSCs genetic engineering. Lentiviral vectors harboring GFP reporter gene allowed for the assessment of LSC transduction efficiency. All used lentiviral vectors showed transduction efficiencies with means of 68.0% ± 13.1%. Transduction efficiencies for each of the vectors used were 74.9% ± 10.9%, 61.7% ± 10.9% and 67.3% ± 15.6% for shNS (non-sense, control LSCs), shβ2M (β2M-silenced LSCs) and shCIITA (CIITA-silenced LSCs), respectively (**Figures 2A, B**).

Colony formation is an *in vitro* characteristic of stem cells associated with their capacity of single cell clonal expansion and their stemness (27, 28). In our study, we observed that transduction with lentiviral vectors did not alter LSCs ability to form colonies. Likewise, LSC growth rates and dynamics were similar in either non-transduced (non-TD) LSCs, or transduced with shNS-, shβ2M- and shCIITA-encoding vectors (**Figure 2C**). Already by day 2 to 3 after seeding on lethally irradiated 3T3 feeder cells, LSCs colonies were already observed; and by day 10 to 11 LSCs were confluent.

### Limbal Stem Cell Phenotypic Markers After Silencing

**Figure 3:**
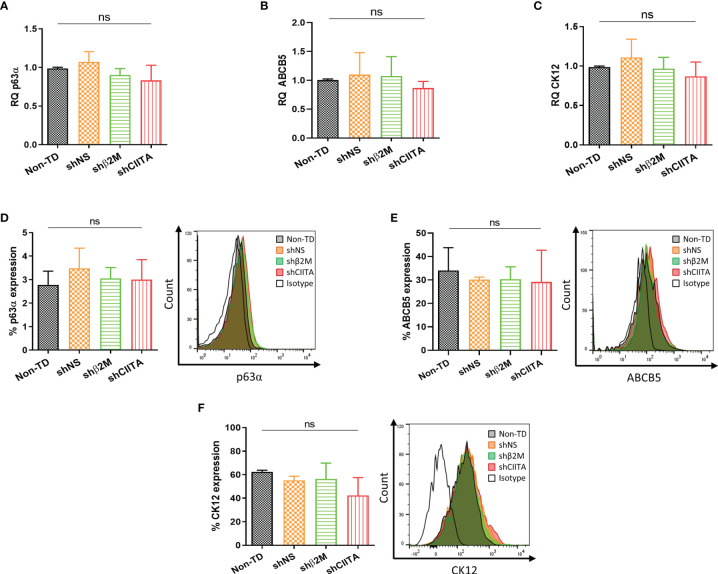
Genetic engineering does not alter Limbal stem cell phenotype. **(A–C)** Transcripts levels of p63α, ABCB5 and CK12 markers of non-transduced LSCs (Non-TD, n=4); non-silenced LSCs (shNS, n=4); β2M-silenced LSCs (shβ2M, n=4) and CIITA-silenced LSCs (shCIITA, n=4). **(D–F)** p63α, ABCB5 and CK12 protein expression remains similar after HLA class I and class II downregulation. Statistical analysis was performed by one-way ANOVA and data are presented as mean ± SD, ns, no significant.

p63α is associated with the proliferative potential of LSCs as well as self-renewal (29). Evaluation of this marker allowed for the quality assessment of the LSCs during cultivation. After genetic engineering, no difference in p63α transcripts levels between non-transduced (non-TD) LSCs, non-silenced LSCs or silenced LSCs (shβ2M or shCIITA) was observed (**Figure 3A**). Furthermore, detection of p63α protein levels showed no differences between the different conditions (**Figure 3D**). In addition, ATP-binding cassette, sub-family B, member 5 (ABCB5) protein was demonstrated to play a role in corneal development and repair (30). In our settings, we observed that frequencies of ABCB5+ LSCs were not significantly altered by silencing HLA expression (**Figures 3B, E**). Furthermore, evaluation of CK12 transcripts and expression levels on cultivated and genetically engineered LSCs remained comparable to those measured in non-modified LSCs (**Figures 3C, F**).

### HLA Class I and II Downregulation on LSCs

**Figure 4:**
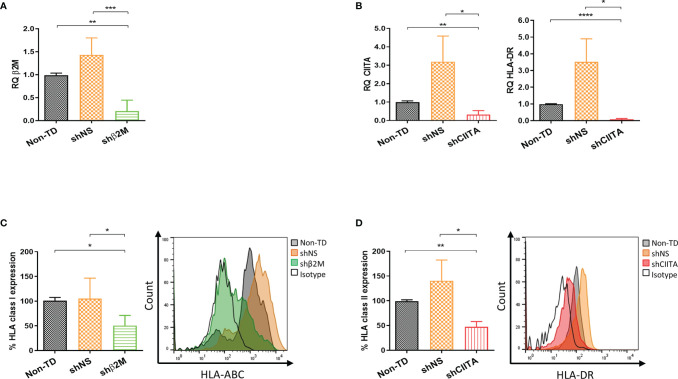
Silencing HLA class I and II expression on limbal stem cells. **(A, B)** Real time analysis for transcripts levels of HLA class I and class II related molecules (β2-microglobulin (β2M), class II major histocompatibility complex transactivator (CIITA) and HLA-DR) of silenced (shβ2M and shCIITA) and non-silenced (shNS and Non-TD) limbal stem cells (n=4) after 48 hours IFNγ (100ng/mL) stimulation. **(C, D)** Mean expression of HLA class I and class II molecules on IFNγ-stimulated limbal stem cells and representative overlay showing HLA class I and class II downregulation effect on transduced cells with shβ2M and shCIITA vectors (n=5). Statistical significance was evaluated by one-way ANOVA and data are presented as mean ± SD, *p < 0.05, **p < 0.01, ***p < 0.001, ****p < 0.0001.

HLA class I molecules maturation and loading depend on β2M and a failure of its expression affects HLA class I presentation on the cell surface. In addition, a disruption or interruption over CIITA expression, which is the master regulator of all HLA class II genes transcription, affects directly their expression (31, 32). LSCs transduced with lentiviral vector encoding for shβ2M and shCIITA resulted in an HLA silencing effect (**Supplementary Figure 2**). Importantly, genetically engineered LSCs after the treatment with IFNɣ (100ng/mL) for 48 hours mimicking a pro-inflammatory microenvironment maintained the downregulation of HLA expression. β2M transcript levels analysis showed a decrease of 80.2% ± 24.1% (p<0.001) compared to the levels detected on non-TD cells (**Figure 4A**). Remarkably, β2M transcript silencing caused a 50.4% ± 20.6% (p<0.01) downregulation on HLA class I surface expression (**Figure 4C**). Comparable results were detected on LSCs transduced with shCIITA-encoding vector. CIITA and consequently HLA-DR transcripts levels were reduced by 68.1% ± 21.9% (p<0.01) and 91.6% ± 9.1% (p<<0.0001), respectively (**Figure 4B**). CIITA downregulation led to a reduction on the expression of HLA class II molecules to 52.6% ± 10.7% (p<0.01) in comparison to LSCs non-TD (**Figure 4D**).

### Silencing HLA Class I and Class II Shows a Protective Effect Against Allogeneic Humoral Response

**Figure 5:**
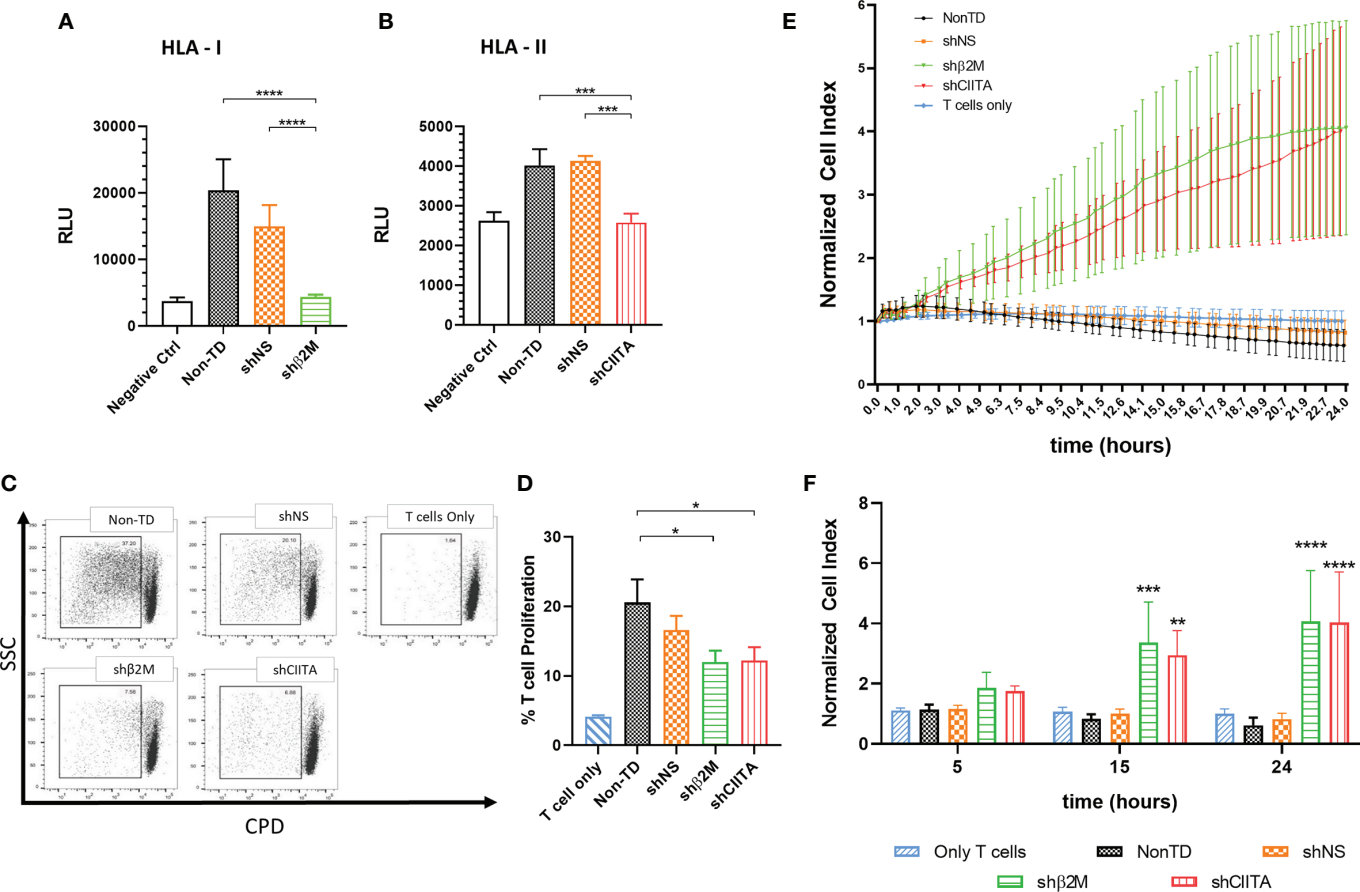
Silencing HLA class I and class II expression shows a protective effect against antibody mediated cellular cytotoxicity and T cell cytotoxicity. **(A)** Antibody-dependent cellular cytotoxicity is reduced during incubation of limbal stem cells with specific antibodies against HLA class I and **(B)** class II molecules, and T effector cells (n=4). **(C)** Representative dot-plots depicting T cell proliferation. **(D)** Bars represents mean percentage of T cell proliferation after 8 days in co-culture with β2M-silenced (shβ2M), CIITA-silenced (shCIITA), non-silenced (shNS) or non-transduced (Non-TD) limbal stem cells (n=6). **(E)** Normalized cell index of silenced (shβ2M and shCIITA) and non-silenced (shNS and Non-TD) limbal stem cells co-culture with pre-primed T cells (n=4). **(F)** Representative time points after addition of pre-primed T cells. One-way ANOVA was used to compare differences between groups and data are presented as mean ± SD, *p < 0.05, **p < 0.01, ***p < 0.001, ****p < 0.0001. RLU, relative luminescence units.

Graft rejection might be triggered by pre-formed or *de novo* donor specific antibodies (33). In order to evaluate the allogeneic humoral response potentially targeting LSCs and the effect of silencing HLA expression, antibody-dependent cellular-mediated cytotoxicity (ADCC) assays were performed. Anti-HLA specific antibodies mediated a significant reduced cytotoxic effect over HLA class I silenced LSCs (4370 ± 336.5 RLU, p<0.0001) compared to non-silenced (14938 ± 3220 RLU) or non-TD (20370 ± 4666 RLU) (**Figure 5A**). Similarly, HLA class II silenced LSCs showed decreased cell lysis rates due to ADCC in comparison to non-silenced (2579 ± 223.2 RLU vs 4128 ± 127.4 RLU, p<0.001) or non-TD (2579 ± 223.2 RLU vs 4019 ± 409.6 RLU, p<0.001) LSCs (**Figure 5B**).

### Silencing HLA Class I and Class II Shows a Protective Effect Against Allogeneic T-Cell Responses

Notably, proliferation of non-primed T cells was significant reduced by the downregulation of HLA class I (11.9% ± 5.7%; p<0.05) or HLA class II (12.2% ± 6.7%; p<0.05) expression on LSCs in comparison to non-TD (20.6% ± 11.5%) or non- silenced cells (16.6% ± 7.2%) LSCs (**Figures 5C, D**).

Furthermore, the strength of primed T-cell cytotoxic responses targeting HLA class I or II-silenced, non-silenced or non-TD LSCs was evaluated. β2M- and CIITA-silenced LSCs showed higher cell survival rates (CI, cell Index) than HLA-expressing control LSCs (Non-silenced and non-TD) (**Figure 5E**). For instance, at 15 hours after initial contact with primed T cells, shβ2M- and shCIITA-expressing LSCs showed a higher CI (3.3 ± 1.3, p<0.001 and 2.9 ± 0.8, p<0.01) in contrast to non-TD or non-silenced LSCs (0.8 ± 0.2 or 1.0 ± 0.1). In contrast to HLA-expressing cells, higher cell survival indexes were maintained over time (24 h) for HLA-silenced LSCs (**Figure 5F**).

### T-Cell Inflammatory Cytokine Response Is Reduced in Presence of Silenced LSC

**Figure 6:**
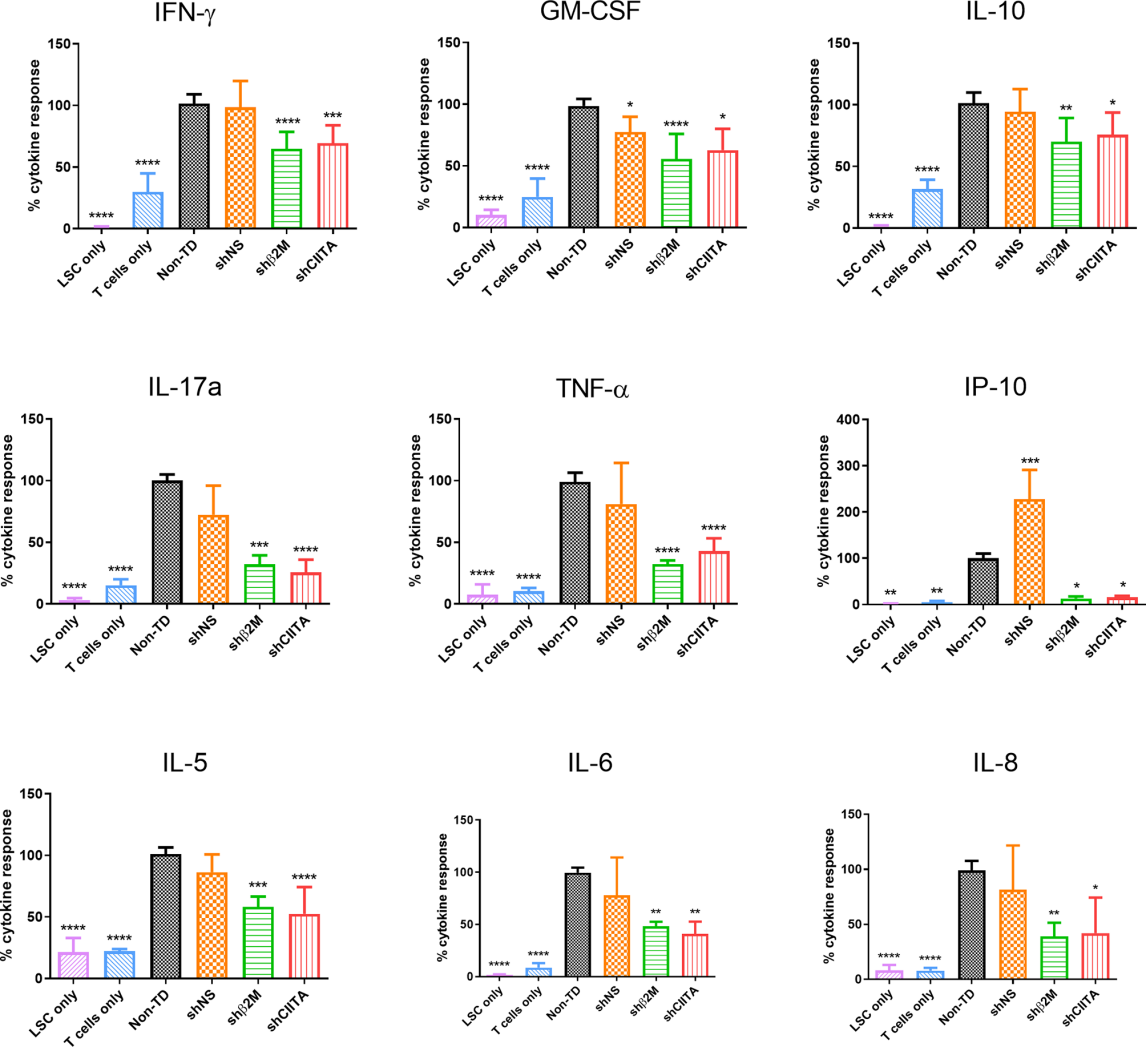
T-cell cytokine response is reduced in presence of HLA silenced limbal stem cells. Percentage of cytokine release (Interferon (IFN)γ, Interleukin (IL)-10, granulocytes-macrophage colony-stimulating factor (GM-CSF), IL-17a, IL-5, IFNγ-induced protein 10 (IP-10), IL-6, IL-8 and tumor necrosis factor(TNF)α by allogeneic T cells co-cultured with non-transduced (Non-TD), non-silenced (shNS) and silenced (shβ2M or shCIITA) limbal stem cells (n=5). Statistical analysis was performed by one-way ANOVA and data are presented as mean ± SD and statistical comparison was done taking Non-TD LSCs as reference. *p < 0.05, **p < 0.01, ***p < 0.001, ****p < 0.0001.

Cytokines and chemokines secreted by T cells are crucial regulators of the immune homeostasis. Increases of pro-inflammatory cytokine levels support graft rejection and failure after transplantation (34, 35). To evaluate the impact of HLA silencing on LSCs in T cell cytokine secretion, allogeneic T cells were co-cultured with silenced and non-silenced LSCs. Higher levels of T-cell cytokine secretion were observed in non-TD and shNS LSC groups. In comparison with non-TD or non-silenced, HLA class I silenced LSCs induced a significantly reduced pro-inflammatory cytokine release [Interleukin (IL)-6 (p<0.01), IL-8 (p<0.01), IFNγ (p<0.0001), TNFα (p<0.0001) and GM-CSF (p<0.0001)]. Moreover, downregulation of HLA class II molecules on LSCs using shRNA-encoding vectors triggered a reduction of IL-6 (p<0.01), IL-8 (p<0.05), IFNγ (p<0.001), TNFα (p<0.0001), GM-CSF (p<0.05) release. Furthermore, IL-17a, which has been described to play a relevant role in cornea transplantation (36) was also reduced [shβ2M-silenced LSCs (p<0.001) and CIITA-silenced LSCs (p<0.0001)] compared to non-TD or non-silenced LSCs (**Figure 6**).

### NK Cell Activity Is Not Affected by HLA Silenced LSC

**Figure 7:**
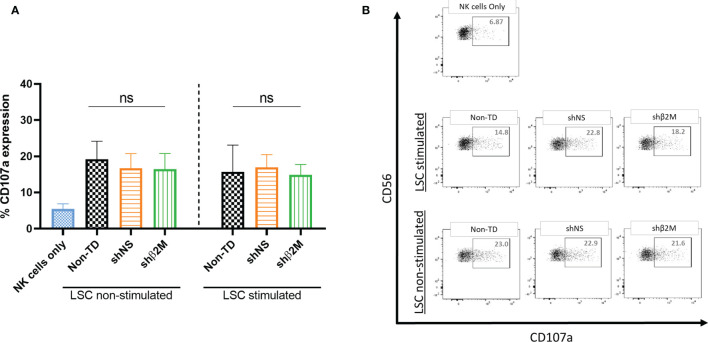
Silencing HLA class I expression on LSCs does not affect NK cell responses. **(A)** Expression levels of the degranulation marker (CD107a) on NK cells co-cultured at target: effector ratio of 1:2 with non-transduced (Non-TD), non- silenced (shNS) and shβ2M-silenced (shβ2M) unstimulated or 48 hours IFNɣ-stimulated limbal stem cells (n=3). **(B)** Representative dot plots of NK cell degranulation measured by the expression of CD107a. Statistical analysis was performed by one-way ANOVA. Data are presented as mean ± SD and comparisons were performed using Non-TD LSCs as reference, ns: no significant.

The activation of NK cells leads to a strong cytolytic effect against their target cells. NK cell cytotoxicity involves the release of granules containing perforins and granzymes and associated with the transient surface expression of lysosomal-associated membrane protein-1 (CD107a) (37). The expression of this molecule on the surface of NK cells has been used as an indirect marker of NK cell cytotoxic function (38). In order to evaluate the effect of HLA class I downregulation on LSCs towards NK degranulation, non-silenced and HLA class I-silenced LSCs were co-cultured with NK cells isolated from healthy donors. The expression of degranulation marker (CD107a) was observed to be similar between Non-TD, shNS and shβ2M LSCs, suggesting that silencing of HLA class I expression on the surface of LSCs does not induce NK cell activation (**Figure 7**). Hence, the residual HLA class I expression on LSCs protect them from being a target for NK cells.

These observations suggest that silenced LSCs are protected against humoral and cellular allogeneic immune responses.

## Discussion

LSCD is a condition leading to loss of visual acuity, photophobia, ocular pain and finally blindness. Emerging strategies to reverse LSCD effects point in the direction of restoring the LSC population on the affected eye either by autologous or allogeneic transplantation (2). In the case of bilateral LSCD, allogeneic transplantation is the only option; however, graft survival after HLA mismatched transplantation is associated with a high risk of rejection (39). Stem cells are described to be hypoimmunogenic due to their reduced expression of HLA class I on their surface and residual or no expression of HLA class II molecules. In contrast to LSCs, their derived keratinocytes are not only able to upregulate HLA class I and II molecules expression, but they might also support the immune system activation (20, 40). In this study, we have shown that LSCs are naturally capable to express high levels of HLA class II protein and they may induce T cell activation and cytokine secretion. These observations indicate that LSCs are immunogenic and might not only be target for antibodies during rejection, but they also can directly elicit and become targets of allogeneic T-cell responses. Furthermore, LSCs might be rejected after allogeneic transplantation due to its natural niche particularities: highly vascularized and the presence of antigen presenting cells like Langerhans cells or macrophages (1, 41, 42). These characteristics facilitate the recipient’s immune system accessibility and activation.

Previously, we showed that silencing HLA expression supports graft survival after allogeneic cell transplantation (21, 43). To reduce the immunogenicity of LSCs, we evaluated the feasibility to silence HLA class I and class II expression in primary cultures of LSCs. Moreover, the impact of silencing HLA expression on allogeneic cellular immune responses was evaluated.

To silence HLA class I and class II expression on LSCs, we used lentiviral vectors encoding for specific shRNAs targeting β2M or CIITA transcripts to mediate RNAi. In terms of regulation of HLA gene expression, RNAi is associated with several advantages in comparison to gene editing technologies such as CRISPR/Cas9 which would cause a complete knockout of HLA expression. In fact, deficiency of HLA expression was previously associated an increased susceptibility to infections (44, 45). RNAi allows cells to express residual levels of HLA. In particular this is crucial to prevent NK cell cytotoxicity and combined with the use of inducible promoters might allow for the re-expression of the gene. Remarkably, recent modifications on CRISPR/Cas9 technologies also offer the possibility to target RNA sequences mimicking the RNAi tool (46).

After LSCs transplantation, inflammation is one of the most common complications (47). Our data suggest that in a pro-inflammatory environment, LSCs might become highly immunogenic due to the upregulation of HLA class I and II expression. LSCs typically form colonies when cultured onto feeder cells and show small cuboidal shape (48). The presence of holoclones is usually a fair indication of good quality of the culture and growth capacity (27, 49). After genetic modification, HLA-silenced and non-silenced LSCs as well as native LSCs showed to have similar morphologies and grow dynamics. Furthermore, the expression of stem cell markers after transduction and HLA silencing indicates for their stemness, which is essential to support the restoration of the LSC population and ensure the maintenance of the corneal epithelium in LSCD eyes after transplantation (50).

Current cultivation techniques focus on maintaining functional LSCs exhibiting good clonogenic capacity as well was high proliferation properties. The use of different scaffolds to mimic LSC niche properties as well as medium supplements to fulfill all nutrients requirements might have an impact on LSCs transplantation outcomes (1, 9, 51). Likewise, any *ex vivo* modification that might be applied to cells needs to be safe, precise, efficient and easily implemented.

The presence of ABCB5+ and p63a+ cells was shown to be required to reverse LSCD (30). After gene modification, no significant differences in the expression of p63α and ABCB5 markers were observed in comparison to control LSCs (non-TD or non-silenced), suggesting that silencing HLA expression did not alter their stemness. In addition, expression of the surface marker ABCB5 after HLA-downregulation might be used to perform an enrichment of therapeutic LSCs, which might have a positive effect on LSCD transplantation outcome (30, 52). Remarkably, the proposed approach to reduce LSC immunogenicity using lentiviral vectors could be easily introduced in the step of cell expansion during the manufacturing process of therapeutic LSCs (52).

In primary LSC cultures, the presence of mature CK12-expressing keratinocytes is expected due to LSCs asymmetric division and spontaneous differentiation. It was previously described that for a successful LSC transplantation the minimum amount of p63α positive cells in the graft should not be below 3% (53). Such percentage, or higher, was reached in all our cultures. Furthermore, our results are in line with observations made in other studies showing that the genetic modification of LSCs does not have a detrimental effect in cell proliferation or stemness. Even though in our approach we did not evaluate stratification of epithelial cells after transduction, it has been described that gene delivery using lentiviral particles does not alter their clonogenic capacity (54–56).

Reduction of HLA class I and class II expression in an allo-transplantation setting may support graft survival as it was already shown in different models (22, 57, 58). In the case of corneal transplantation or LSCs transplantation, systemic immunosuppression is used specially in high-risk patients. Severe side effects associated with systemic immunosuppression remain a relevant concern (59, 60). Thus, strategies to decrease graft immunogenicity might become an alternative to the immunosuppressive therapy supporting the patient’s quality of life.

LSC niche is a highly vascularized area where humoral and cellular recipient responses may trigger a rejection process against allogeneic LSCs (61). After transplantation, development of *de novo* donor specific antibodies (DSA) against donor HLA molecules leads to graft loss of function and rejection supported by the complement system as well as macrophages, natural killers or T cells (33, 62). Even though in cornea transplantation the production of alloantibodies does not correlate with rejection, antibody-dependent cytotoxicity may still occur and increases the risk for graft failure (63). Indeed, antibody-mediated keratolimbal allograft rejection has been observed and its treatment with intravenous immunoglobulin (IVIG) showed to be effective in blocking rejection process (62). In our study, we observed that HLA-silenced LSCs were protected from antibody-mediated cellular cytotoxicity in comparison to fully HLA-expressing LSCs. In previous studies, we have demonstrated that HLA-silenced cells including Megakaryocytes or endothelial cells are protected from antibody-mediated complement-dependent cytotoxicity (24). Similarly, evaluation of the direct response of alloreactive T cells towards silenced LSCs showed lower rates in T cell proliferation and cytotoxicity. These decreased responses demonstrate that HLA-silenced LSCs can also escape specific allogeneic T-cell responses. Therefore, silencing HLA expression in LSCs confers them an “invisibility cloak” (21). Survival of LSCs is a key to support the corneal epithelium repopulation and homeostasis as well as to reestablish stem cell population serving as a barrier for corneal conjunctivalization, which is typical in LSCD (2).

Pro-inflammatory cytokines are able to transiently inhibit wound healing and support rejection (64). The prolonged exposition of LSCs to pro-inflammatory factors has a negative impact on LSCs survival and accommodation after transplantation due to their effect over LSCs morphology, cell cycle and colony-forming efficiency (64). Additionally, pro-inflammatory cytokines play a role in immune system activation and support orchestration of rejection process reducing graft survival (65, 66). In allogeneic settings, activated T cells mediate allograft rejection by releasing pro-inflammatory cytokines. In our model, we observed that HLA-silenced LSCs induced a significant reduced T cell cytokine secretion. Interestingly, production of IL-17a, a pro-inflammatory cytokine and a relevant factor in cornea transplantation, was also observed to be reduced. Presence of IL-17a in corneal tissue has a role in its immune privilege and may be involved in allograft survival (67, 68). However, IL-17a might indirectly support neutrophils survival and tissue infiltration in allo-transplantation settings (69). Activated neutrophils can trigger rejection by recruiting CD8+ T cells through FAS ligand expression. Furthermore, they might be able to interact with B cells and induce antibody-mediated rejection (70).

NK cytotoxicity is regulated by activating and inhibitory signals through different receptors on their surface. The interaction of HLA-specific inhibitory receptors on NK cell surface with their ligands (HLA) provides enough negative signals to avoid NK cell activation. In fact, cells that express sufficient amount of HLA class I on their surface are protected from NK cytotoxic attack (71). On the contrary, it has been observed that the lack of HLA class I molecules triggers NK cell activation. In previous studies, our group has demonstrated that a residual expression of HLA class I is sufficient to prevent NK cell activation (58, 72). Here, we observed that the residual expression of HLA class I on LSCs is enough to provide NK cells sufficient inhibitory signals prevent activation and cytotoxicity.

Our findings are a proof-of-concept of the feasibility of generating low immunogenic LSCs with the capability to escape allogeneic humoral and cellular immune responses.

## Conclusion

In summary, our study shows that under inflammatory conditions LSCs are highly immunogenic and capable to trigger immune cell cytotoxic responses. Reduction of LSCs immunogenicity by silencing HLA expression may provide a great advantage to prevent rejection and prolong graft survival. We demonstrated that LSCs are able to upregulate HLA molecules in a pro-inflammatory microenvironment, which may lead to rejection after allogeneic transplantation. HLA-silenced LSCs maintained the typical morphology, phenotype and *in vitro* proliferative properties after silencing HLA expression. Silencing HLA expression on LSCs conferred protection against antibody-mediated cellular dependent cytotoxicity. Remarkably, T-cell proliferation, cytokine release and cytotoxicity were significantly decreased in cultures using HLA class I or class II-silenced LSCs. Pro-inflammatory cytokine secretion such as IFNγ, IL-6, IL-8, TNFα and IL-17a was also reduced, suggesting a weaker alloimmune response induced by HLA-silenced LSCs compared to fully HLA-expressing LSCs. The use of low immunogenic LSCs may offer an opportunity to improve graft survival after LSC allotransplantation.

## Data Availability Statement

The original contributions presented in the study are included in the article/**Supplementary Material**. Further inquiries can be directed to the corresponding author.

## Author Contributions

EV conception and experimental design, analysis of the data, wrote the manuscript. MC collection of data and final approval of manuscript. MBö AS, NH, MBe, CB, RB, and SF provide essential study material, assistance in experiments and final approval of manuscript. CF conception of the study, data analysis and wrote the manuscript. All authors contributed to the article and approved the submitted version.

## Funding

This study was supported by the Excellence Cluster REBIRTH (EXC62, Unit 6.3).

## Conflict of Interest

The authors declare that the research was conducted in the absence of any commercial or financial relationships that could be construed as a potential conflict of interest.

## Publisher’s Note

All claims expressed in this article are solely those of the authors and do not necessarily represent those of their affiliated organizations, or those of the publisher, the editors and the reviewers. Any product that may be evaluated in this article, or claim that may be made by its manufacturer, is not guaranteed or endorsed by the publisher.
