# Supplementary material for: Genetic Modification of Limbal Stem Cells to Decrease Allogeneic Immune Responses
Source: Front Immunol. 2021 Dec 9;12:747357. doi: 10.3389/fimmu.2021.747357 (PMC8696204; doi:10.3389/fimmu.2021.747357)
Supplement: Supplementary file 1 [file DataSheet_1.docx]

Genetic modification of Limbal Stem Cells to decrease allogeneic immune responses

Emilio Valdivia^1^, Marina Bertolin^2^, Claudia Breda^2^, Marco Carvalho Oliveira^1^, Anna Katharina Salz^3^, Nicola Hofmann^3^, Martin Börgel^3^, Rainer Blasczyk^1^, Stefano Ferrari^2^, Constanca Figueiredo^1*^

**Supplementary Material**


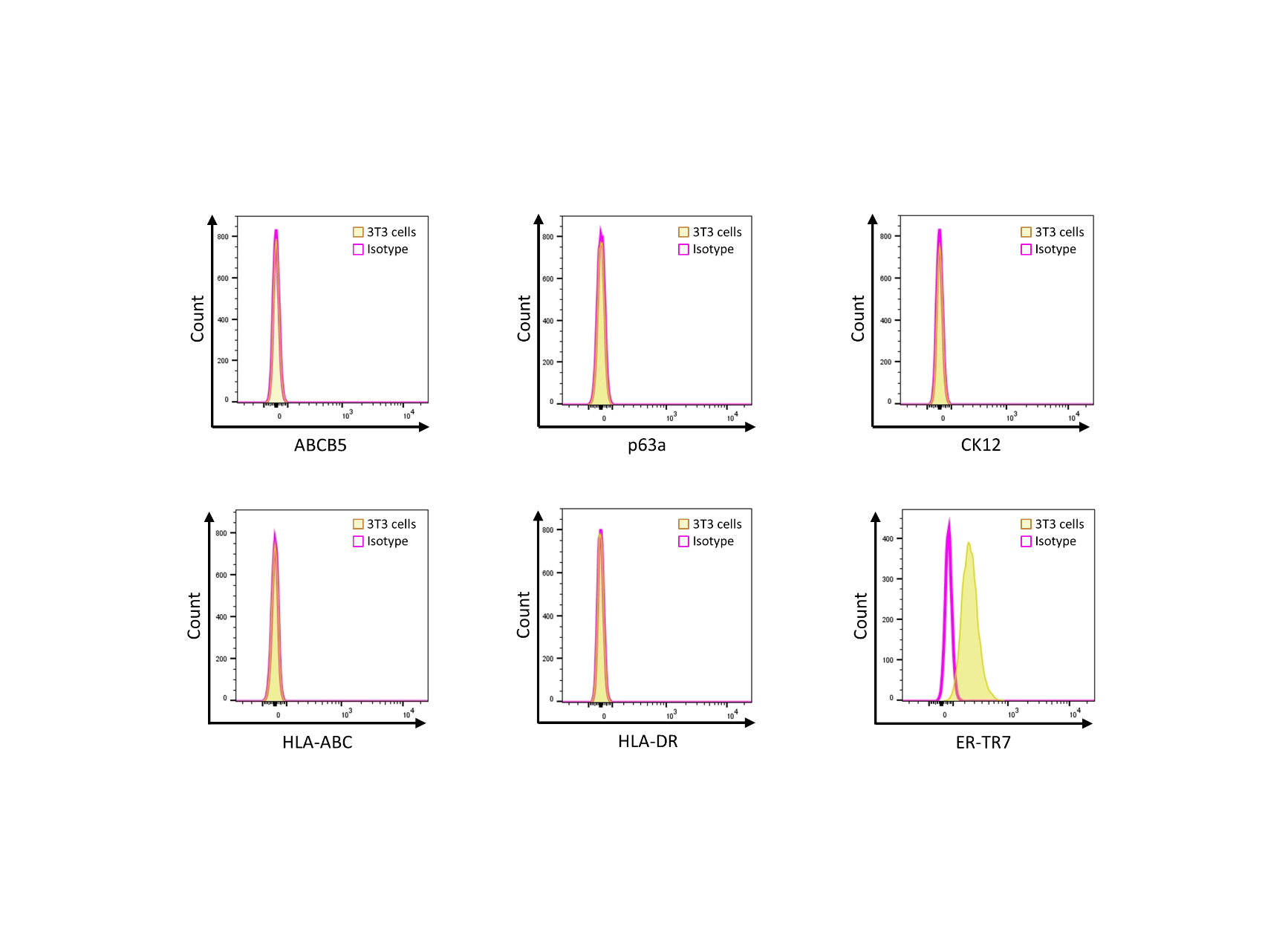


**Supplementary Figure 1.** Cross-reaction tests for human specific antibodies on 3T3 feeder cells. Specific antibodies used to identify and characterize limbal stem cells were analyzed for cross-reaction activity on 3T3 feeder cells. Human anti-HLA-ABC PE-conjugated (clone W6/32; Biorad, California, USA), anti-HLA-DR APC/Cy7-conjugated (clone L243; Biolegend, California, USA), unlabeled anti-ABCB5 (polyclonal; Thermo Fisher Scientific) combined with PE-conjugated secondary antibodies were tested on feeder cells using identical protocol as followed for limbal stem cells analysis. Intracellular staining was performed using IntraPrep permeabilization reagent (Beckman Coulter, Krefeld, Germany), anti-p63α (clone I504; Abbexa, Cambridge, UK) and CK12 (clone EPR17882; Abcam, Cambridge, UK) primary antibodies combined with PE- and APC/Cy7- conjugated secondary antibodies, respectively. Moreover, specific fibroblast marker (clone ER-TR7; Santa cruz biotechnology, Texas, USA.) was used to identify 3T3 feeder cells.


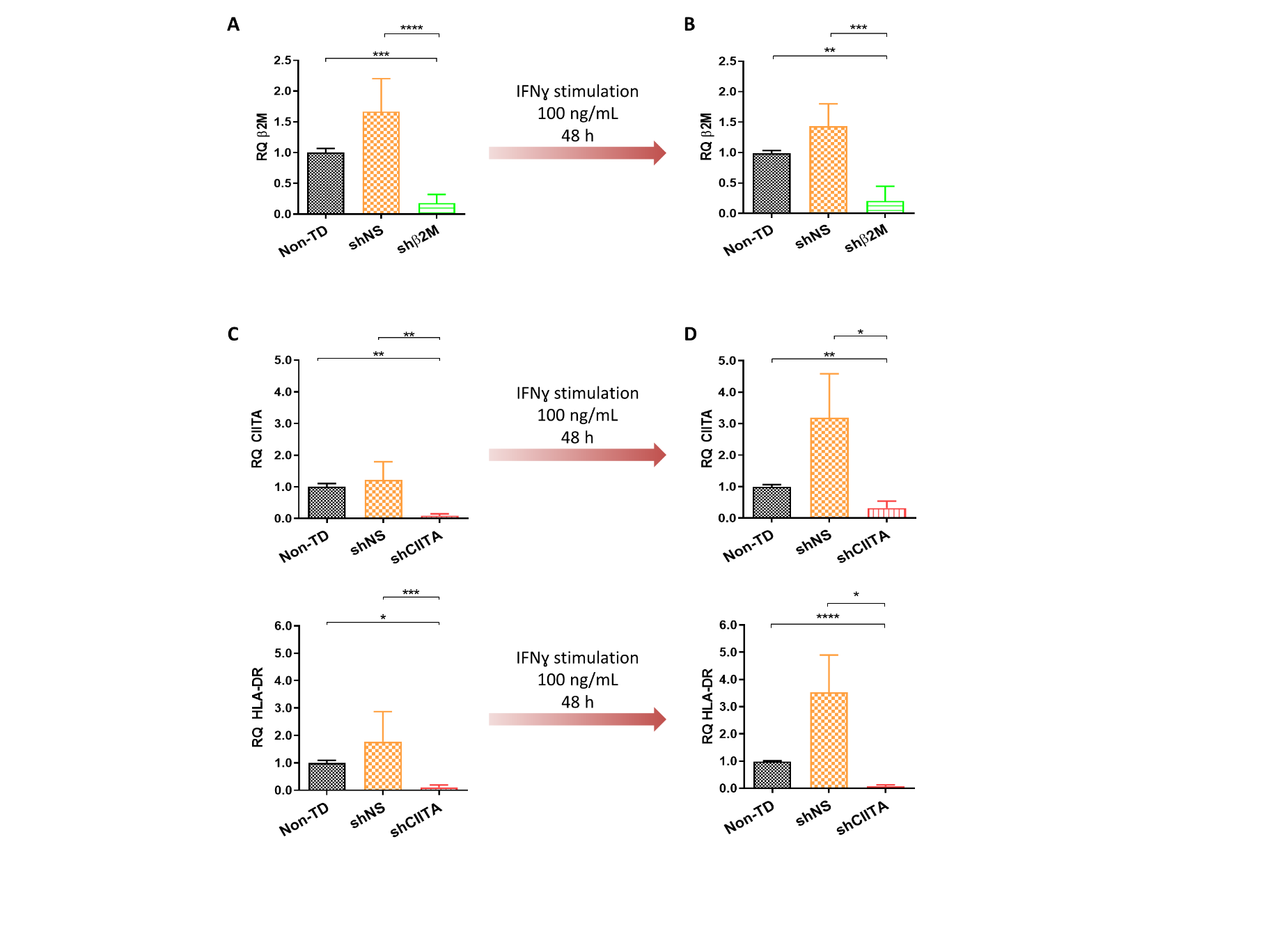


**Supplementary Figure 2.** Silencing effect in LSCs using lentiviral vectors encoding for shRNA targeting β2-microglobulin (β2M) and class II transactivator (CIITA). **(A)** Relative quantification of β2M expression on LSCs non transduced (Non-TD), transduced with lentiviral vector encoding for non-sense shRNAs (shNS) and shRNAs targeting β2M (shβ2M)(n=4). **(B)** β2M downregulation is maintained even after LSC stimulation with IFNɣ (n=4). **(C)** Transcripts levels of CIITA and HLA-DR detected on Non-TD LSC, shNS- and shCIITA- expressing LSCs (n=4). **(D)** Reduced levels of CIITA and HLA-DR expression remained after stimulation with IFNɣ (100 ng/mL) for 48 hours (n=4). Non-TD LSCs and transduced with a lentiviral vector encoding for non-sense shRNAs (shNS) were used as controls. Statistical significance was evaluated by one-way ANOVA and data are presented as mean ± SD, *p<0.05, **p<0.01, ***p<0.001, ****p<0.0001
